# Localizing Genes to Cerebellar Layers by Classifying ISH Images

**DOI:** 10.1371/journal.pcbi.1002790

**Published:** 2012-12-20

**Authors:** Lior Kirsch, Noa Liscovitch, Gal Chechik

**Affiliations:** The Gonda Multidisciplinary Brain Research Center, Bar-Ilan University, Ramat Gan, Israel; Duke University, United States of America

## Abstract

Gene expression controls how the brain develops and functions. Understanding control processes in the brain is particularly hard since they involve numerous types of neurons and glia, and very little is known about which genes are expressed in which cells and brain layers. Here we describe an approach to detect genes whose expression is primarily localized to a specific brain layer and apply it to the mouse cerebellum. We learn typical spatial patterns of expression from a few markers that are known to be localized to specific layers, and use these patterns to predict localization for new genes. We analyze images of in-situ hybridization (ISH) experiments, which we represent using histograms of local binary patterns (LBP) and train image classifiers and gene classifiers for four layers of the cerebellum: the Purkinje, granular, molecular and white matter layer. On held-out data, the layer classifiers achieve accuracy above 94% (AUC) by representing each image at multiple scales and by combining multiple image scores into a single gene-level decision. When applied to the full mouse genome, the classifiers predict specific layer localization for hundreds of new genes in the Purkinje and granular layers. Many genes localized to the Purkinje layer are likely to be expressed in astrocytes, and many others are involved in lipid metabolism, possibly due to the unusual size of Purkinje cells.

## Author Summary

The way gene expression is spatially distributed across the brain reflects the function and micro-structure of neural tissues. Measuring these patterns is hard because brain tissues are composed of many types of neurons and glia cells, and average gene expression across a region mixes transcripts from many different cells. We present here an approach to identify genes that are primarily expressed in specific brain layers or cell types, based on analyzing high resolution in-situ hybridization images. By learning the spatial patterns of a few known cell markers, we annotate the expression patterns of hundreds of new genes, and predict the layers and cell types they are expressed in.

## Introduction

A key problem in current neuroscience is to characterize how the transcriptome governs the structure and function of the brain [1]. The challenge is particularly hard in the mammalian central nervous system because every brain region contains numerous types of neurons, astrocytes, and other non brain-specific cells such as blood vessels and immune cells. Each of these cell types have their own molecular profile, and typically exhibit unique patterns of gene expression [1], [2]. These patterns may depend not only on the individual cells, but also on their interaction with neighboring cells. Cell-specific expression patterns determine the formation of both the microcircuitry and the long-range neuronal connections through specific molecules [3]. These patterns also shape the functional properties of neurons and glia. Understanding the molecular basis of brain function therefore requires dissecting gene expression patterns into their cell-specific and layer-specific components.

Unfortunately, measuring layer-specific expression is costly and time consuming, and as a result, only a few such datasets have ever been collected [4]–[8]. Cell-type specific data can be collected by growing cell cultures in vitro, which may differ from natural growth conditions, or by sorting cells using known markers [6]–[8]. It is also possible to collect cells from specific cortical layers using laser microdissection [5]. Alternatively, in some cases it is possible to profile the transcriptome of strains that lack a specific type of cells, and compare them to normal developing animals [9].

Here we propose another approach, based on machine vision, to identify layer-specific genes. The method is based on modeling the spatial expression patterns observed in *in-situ hybridization* (ISH) images of a few genes that are known to be expressed exclusively in specific layers (cell-type markers). Using the learned patterns, we then automatically scan the genome-wide ISH database and detect all other layer-specific genes.

**Figure 1:**
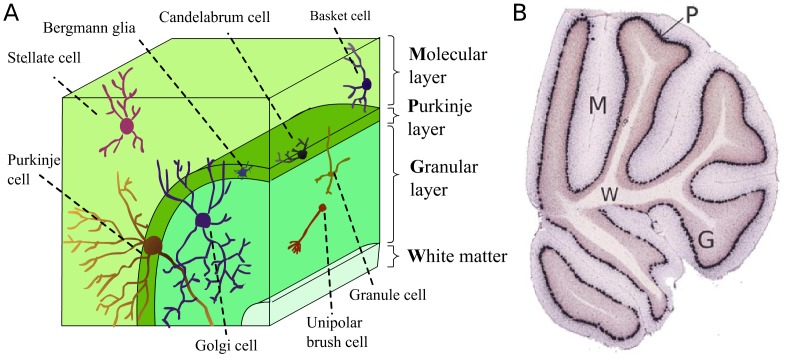
Cerebellum layers and cell types. (**A**) Cell types and their location across the cerebellar cortical layers. (**B**) Cerebellum ISH image of Calb1. The different layers can be easily discriminated. P - the Purkinje layer; G - the granular layer; M - the molecular layer; W - the white matter.

The current paper focuses on the cerebellum, which has been extensively studied due to its highly organized laminar structure. The cerebellum contains three cortical layers and a white matter layer (Figure 1). The innermost cortical layer is the *granular layer*, a densely packed layer containing mossy fibers, the cell bodies of granule cells, uni-polar brush cells, and Golgi cells. The middle cerebellar layer is the *Purkinje layer*, containing the cell bodies of the Purkinje cells, Candelabrum interneurons and Bergmann glia. The third, outermost, cortical layer is the *molecular layer*, containing the dendritic arbors of the Purkinje cells and the inhibitory Stellate and basket interneurons. These three cerebellar cortical layers and cell types are illustrated in Figure 1 A. Finally, the white matter layer resides within the inner most part of the cerebellum. For a more detailed review of cerebellar cell types see [10].

We use the large-scale collection of in-situ-hybridization images of the cerebellum collected by the Allen Institute [11], together with a small list of genetic markers of cell types that are known to reside in specific cerebellar layers. To achieve high classification accuracy, we combine multiple images of each gene. Each ISH image is represented using histograms of *local binary patterns* (LBP) [12] that are collected at multiple resolutions. This representation captures characteristic spatial structures at multiple scales and improves accuracy significantly over a single-resolution representation.

When the trained classifiers are evaluated on a held-out data of similar markers, they correctly classify each of the four main cerebellum structures with more than 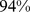 accuracy (AUC). Furthermore, when applied to the full mouse genome, manual inspection of the 250 top predictions of each class shows that the classifiers successfully identify localized genes. Overall, we identify 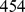 genes localized primarily to the Purkinje layer, 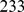 genes in the granular layer, and several new layer specific markers for the white matter and the molecular layer. Some of these genes were previously proposed as layer-specific markers, but hundreds of the genes detected have never been associated with these layers.

## Results

This paper is organized as follows. We start by describing the ISH image dataset and discuss our machine vision approach for classification. Next, we describe cross-validation experiments that evaluate the accuracy of the trained classifiers. We then analyze the properties of new layer-specific predictions, also during brain development. Finally, we compare the predicted layer-specific genes to previous literature, and discuss in details two new examples of such genes, Fam107b and Map2k6.

### Expression patterns measured using in situ hybridization

Our approach is based on learning spatial gene expression patterns measured using non-isotopic ISH. ISH is used to localize RNA expression of a target gene in a tissue. We used ISH data collected by the Allen Brain Atlas (ABA) [11] available at http://mouse.brain-map.org. ISH measurements were gathered for the adult mouse full genome, covering 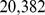 genes. For each gene, mice brains were dissected into slices in sagittal and coronal sections, and the slices were fluorescently labeled and imaged to reveal places where the gene is expressed. The outcome of this process is a set of images showing the gene expression pattern across the whole mouse brain in high spatial resolution.

**Figure 2:**
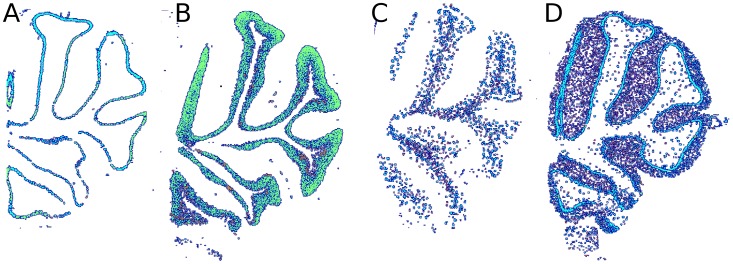
Masked ISH images of the mouse cerebellum from the Allen Brain Atlas. Each image shows expression of a different gene, highlighting cells located at a different layer. Images are based on ISH measurements from ABA, but shown using our own color map for better visualization in print. (**A**) Calb1, expressed in the Purkinje layer. (**B**) Neurod1, expressed in the granular layer. (**C**) Plp1, expressed in the white matter. (**D**) Gad1, expressed in the molecular and Purkinje layers.

This paper focuses on four distinct layers of the mouse cerebellum, each layer contains a different set of neurons and glia cells. Figure 2 A–D shows examples of sections from the mouse cerebellum, stained with four different markers. The leftmost panel depicts the expression of Calbindin1 (Calb1), a well known marker of Purkinje cell bodies, Figure 2A. Purkinje cells are known to be organized in the thin Purkinje layer, and indeed, the Calb1 expression forms a distinct spatial pattern in the form of a thin stripe. Other layers of the cerebellum can also be observed: Figure 2B shows the expression of Neurod1, a known marker of granular cells that reside in the granular layer; Figure 2C shows the expression of Plp1, a myelin proteolipid protein marking cells which reside in the underlying cerebellum white matter; Figure 2 D shows the expression of Gad1, which is expressed in the molecular layer and also in the Purkinje layer. The molecular layer contains the dendritic arbors of the Purkinje cells, whose bodies lie in the Purkinje layer (Figure 1A). As a result, most genes expressed in the molecular layer are also expressed in the Purkinje layer. We therefore defined a class that contains genes which show expression in the molecular layer and also in the Purkinje layer.

While a few dozen genes are already known to be specifically expressed in particular cell types and cerebellar layers, such location information is still unknown for most of the mouse genome. Here we use these few genes that are known to mark a specific cerebellar layer, learn their spatial expression pattern and predict new localization information for many genes. We train a separate binary classifier for each of the four classes: Purkinje, granular, molecular and white-matter. The molecular class includes genes that are expressed both in the molecular and in the Pukinje layer, but we name this class *molecular* for simplicity. The next section discusses the representation of the ISH images used as input to these classifiers.

### Representing ISH images

**Figure 3:**
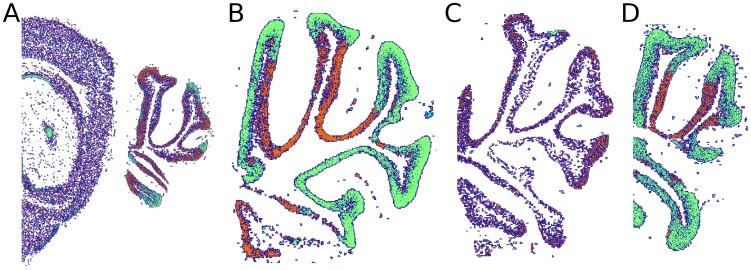
Examples of the variability within a single layer. Shown are four genes which exhibit a high expression in the granular layer but not in the other layers. Notice the difference in texture, size, position, expression level (color) and structure shape. (**A**) Fxr2h. (**B**) Calb2. (**C**) Kcnj3. (**D**) Kcnk1.

For natural images, there has been extensive research on extracting features that are useful for object recognition and detection [12]–[16]. However, for ISH images of complex tissues, only little work has been done on developing such discriminative features. ISH should not be confused with single-cell FISH image analysis, which aims to identify subcellular structures. Most existing work on detection and classification of ISH tissue expression images focused on gross anatomy, where the global shape plays a prominent role. [17]–[21]. These methods, which employ advanced machine vision techniques and achieve state of the art results, depend on a pre-processing stage in which the images are normalized and registered. For example, [17] used pyramid kernels to identify expression patterns in fly embryos and [20] have tested a series of techniques with images that were transformed to a standard shape size and orientation. Such standardization is feasible with fly embryos whose shape is largely regular, but for brain layer recognition such standardization poses new challenges as shown below in Figure 3. As a result, the question of selecting a good representation for analyzing ISH expression images of brains is still open.

Since images of different genes were taken from different mouse brains, and brains differ considerably in their detailed anatomy, spatial expression patterns of the same layer vary considerably across brains. Figure 3 shows four genes expressed in the granular layer, illustrating the variability in size and shape across individuals. As a result of these differences, naive approaches that use voxel-to-voxel spatial correlations between images [22] often fail to match images of the same layer. This problem is particularly difficult with the cerebellum with its elongated structures that are sensitive to small shifts of the images. A good representation of an expression image should therefore be invariant to the types of distortion found across brains.

**Figure 4:**
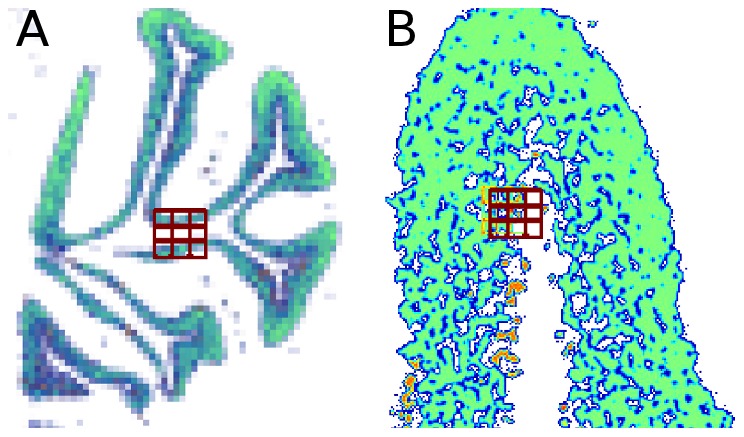
Structure and texture are captured using different spatial resolutions. (**A**) A low resolution ISH image of a granular layer gene from Figure 2 B. The 3×3 LBP grid capture the gross finger-like structure. (**B**) A high resolution version (zoomed to scale) of the same image. The 3×3 LBP grid captures texture patterns.

An important aspect of layer-specific expression patterns, is that they exhibit structures at multiple scales. At the coarse scale, the gross structure of the cerebellum contains “finger-like” structures, each having a width of a few millimeters (Figure 4 A). At the same time, genes expressed in different layers may also lead to different “textures” that can be observed at a more refined resolution (Figure 4 B). The texture is determined by the particular spatial distribution of the cells in which the genes are expressed. To take advantage of all these sources of information, here we analyze each image at multiple scales, by down-sampling each image before extracting features. Analysis at multiple scales has been used in many other applications, such as texture classification [23], and we show below that combining multiple resolutions improves classification accuracy in the current problem as well.

At every scale, we represented an image using a histogram of local binary patterns (LBP, [12]), together with the mean intensity of the image. We then combine the feature vectors from the different scales into a single representation. This representation captures both fine texture and coarser structures. For example, applying LBP to a coarsely sampled image as in Figure 4 A captures large structures, while applying LBP to a high resolution version of the same image can capture refined texture statistics as shown in Figure 4B. All four cerebellar layers contain recurring patterns at multiple scales.

### Two-level classification: image and gene classifiers

In our data, each gene is associated with multiple ISH images, collected from different brain slices and possibly multiple brains (typically 2–8 images per gene). Our task is to assign a cerebellar layer to each gene, rather than to classify individual images. Therefore, we need to combine classification of images into a single unified decision at the level of a single gene. Common approaches to combine scores from multiple patches, include *max-pooling* and *average pooling* [24]. Here we take a discriminative approache to solve this task. First, we train a classifier operating on labeled images, and then classify each gene by combining the scores provided by the image classifier.

We compared three different ways to combine image scores into a decision over genes (*Mean image score*, *Mean LBP features* and *Two-level classifier*). We also tested a fourth approach as a baseline, treating each image independently, and making a prediction at the level of single images.

**Figure 5:**
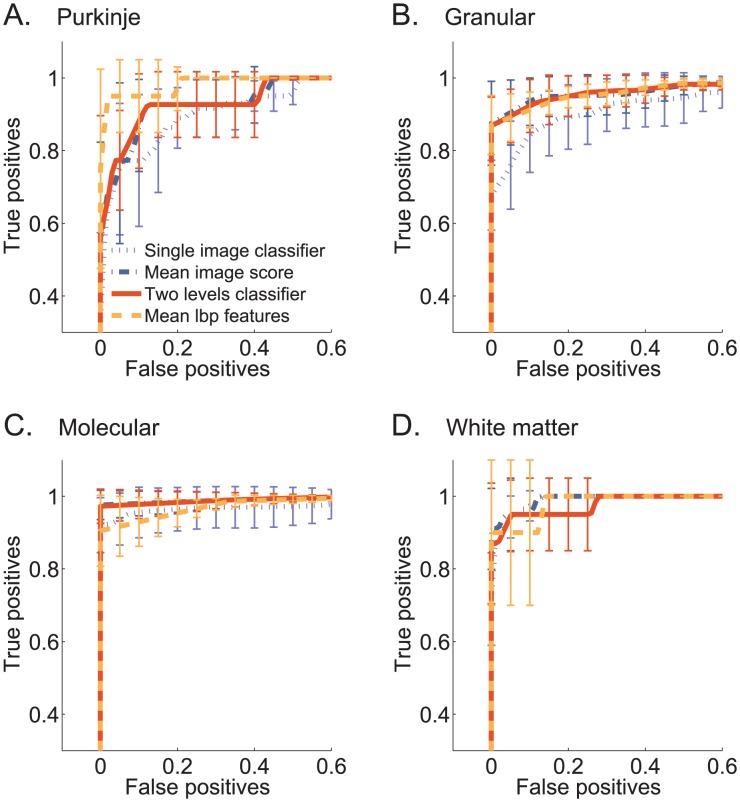
Classifier results for each cerebellar layer. ROC curves comparing classifiers for the four layer classification tasks. For each layer, results are shown for four classifiers. Shown are results for *Single image classifier* (dotted purple line), *Mean image score* uses average pooling (dash-dotted blue line); *Two levels classifier* learns weights over pooling statistics using SVM (solid red line); *Mean LBP features* (dashed yellow line), uses the average LBP vector for each gene. Error bars denote the standard deviation across five cross-validation folds. Results for the two layers classifier: (**A**) Purkinje vs random set. AUC = 0.949
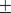
0.02. (**B**) Granular vs random set. AUC = 0.96
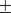
0.017. (**C**) Molecular vs random set. AUC = 0.987
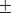
0.011. (**D**) White matter vs random set. AUC = 0.983
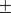
0.013.

Figure 5 shows evaluations of the precision of these four approaches, computed on held-out data. Each panel shows the ROC curve for a different layer, where the classifiers were trained to detect each cerebellar layer from a randomly selected set of genes. All classifiers achieve a very high area under the ROC curve (AUC 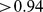 for all four categories). This shows that the two-level approach succeeds to combine information from multiple images.

We further trained classifiers to discriminate between every two classes (“one *vs.* one”). Table 1 shows that most classes can be easily discriminated, except the pair molecular *vs.* granular, which are confused 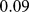 of the time.

**Table 1:** All-to-all class confusion matrix (1-AUC).

Error (1-AUC)	Purkinje	Granular	Molecular	White matter	Negative set
Purkinje	-	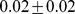	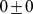	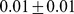	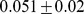
Granular		-	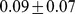	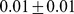	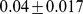
Molecular			-	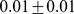	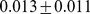
White matter				-	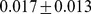

Classification errors (1-AUC) for classifiers trained for one class against another class. All pairs can be discriminated with high confidence except granular and molecular layer genes which are confused 9% of the time.

**Figure 6:**
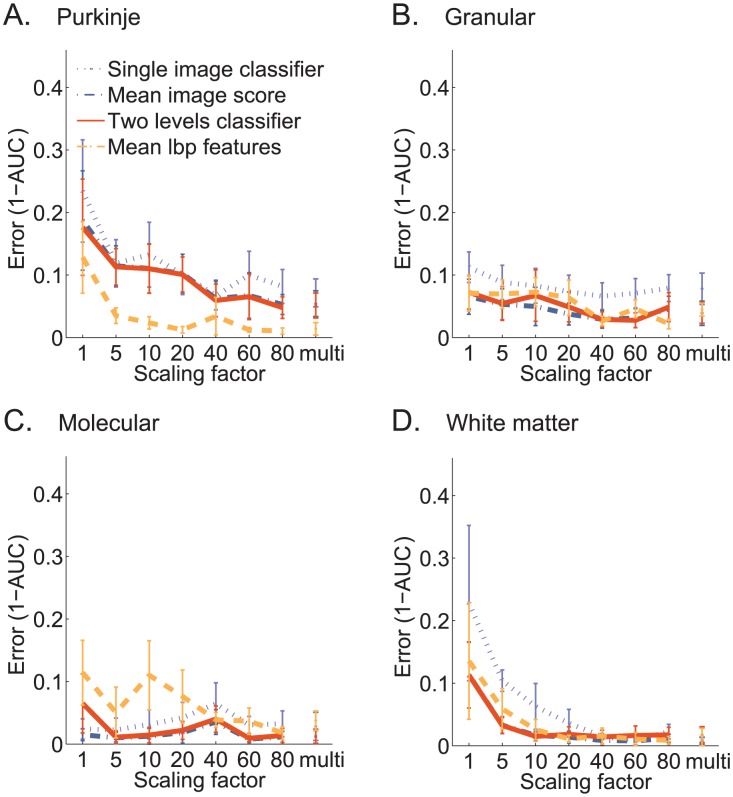
The effect of image resolution on classification accuracy. We scaled images by a factor of 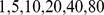, where a value of 10 means that all pixels in a 10×10 patch (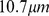 by 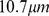) were averaged into one pixel. Four classifiers are compared, one for each layer, as in Figure 5, but this time plotting the AUC for various downsampling factor levels. Using multiple resolutions (rightmost value) consistently improves over using a single resolution. (**A**) Purkinje *vs* random set. (**B**) Granular *vs* random set. (**C**) Molecular *vs* random set. (**D**) White matter *vs* random set.

To further evaluate the effect of image scale on classification accuracy, we compared the accuracy obtained using model trained at different scales (Figure 6). We also tested a model that uses multiple scales. Using a multi-scale representation consistently achieves higher (or similar) AUC than using any single scale. Overall, coarse resolutions perform better. Surprisingly, the granular layer and also the molecular layer can be discriminated accurately using high resolution images (Figure 6B,C). The high accuracy obtained when using fine-resolution features suggests that the granular and molecular layers contains texture patterns that are useful for discrimination from other layers even if their coarse expression patterns are similar.

We compared our detection results with results obtained using the ABA NeuroBlast [22] http://mouse.brain-map.org/. NeuroBlast ranks genes according to their spatial correlation to a target gene. First, every in-situ image is registered to a reference template and then the spatial correlation between the registered and normalized images is computed. NeuroBlast provided a good AUC for the Purkinje layer (0.82) and the granular layer (0.73). However, NeuroBlast was less successful in detecting genes expressed in the molecular layer (0.34). For the white matter, a high AUC is obtained (0.98) and this is possibly due to the way we manually constructed labeled set of genes. Since we could not find many markers that are primarily expressed in the white matter, we used NeuroBlast to find genes that have white matter expression. NeuroBlast suggested genes which have a close expression pattern to the few known literature white matter markers. We then manually validated these suggestions. More information on the way the dataset was constructed is found in the Methods section.

Registration based approaches, such as ABA NeuroBlast, can be sensitive to small shifts and such shifts are prevalent when aligning images of different brains. For example, a shift of a few microns can cause the thin line of the Purkinje layer to misalign with the target image, leading to a low correlation. Our registration free approach is less sensitive to such misalignments.

### Genome-wide predictions

The above results showed that the trained classifiers achieve high accuracy on held out data of known markers. We further applied the trained layer predictors to the full mouse genome (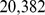 genes in the ABA database). All predictions, including lists of genes that are localized to specific layers are available online at http://chechiklab.biu.ac.il/~lior/cerebellum.html. Out of 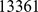 genes that are expressed in the cerebellum, 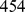 genes are predicted to be primarily expressed in the Purkinje layer, 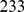 in the granular layer, 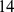 in molecular layer and 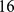 in the white matter.

We validated the predictions by manually scanning the top predicted genes (and the bottom predicted genes - Table 2), visualizing their measured expression patterns, and comparing them to the patterns expected at that layer. Out of the top 250 genes predicted to be localized to the Purkinje layer we correctly classified 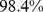. Similarly, 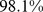 of the top 250 granular layer prediction were accurate. The precision was worse for localization of the molecular layer: All 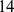 prediction had a molecular expression, but 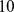 out of the 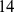 also had a granular expression. Finally, 10 out of 16 predicted white matter were positive. It should be clarified however, that many of the genes that exhibited localized expression in one cerebellar layer, are also expressed in other regions of the brain, sometimes very widely. Also, despite the fact that most of the training images in the molecular class show expression in the molecular layer and also in the Purkinje layer, our classifier was able to identify genes that show expression only in the molecular layer.

**Table 2:** Classification results for the bottom 100 genes.

	Granular layer	Granular and Purkinje layers	Purkinje layer	Purkinje layer scattered expression	Molecular layer	Molecular and Purkinje layers	Cerebellum scattered expression
Purkinje layer detector	7	6	1	8	0	0	78
Granular layer detector	7	8	0	6	1	1	66

We manually examined the 100 lowest scoring genes predicted by the Purkinje layer detector and the granular layer detector. The table shows the classification of the lowest scoring genes into 7 observed categories.

Applying the white-matter classifier and the molecular layer classifier to the full genome yielded very few positively scored genes. This could be attributed to the small number of positive samples in the training set for these classes. Indeed, when we manually examined one thousand of the genes in the database we only found one gene that was exclusively localized to the white matter (and one gene localized to the molecular layer). In comparison, there were many more genes localized to the granular or the Purkinje layers.

To find out if our classifiers can be generalized, and can detect genes on images that are different in their spatial expression organization. We applied the four classifiers that were previously trained on the sagittal sections to all the images available in coronal sections, covering 4000 genes. Unlike sagittal section images, many coronal images contain parts of the brain from outside the cerebellum and their layer organization is quite different from the sagittal images. Despite these large differences between the training set and the test set, the Purkinje layer classifier generalized well and detected genes that are primarily expressed in the Purkinje layer in 95% of the top 100. The other layer detectors did not generalized as well. Results are available at http://chechiklab.biu.ac.il/~lior/cerebellum.html

### Characterizing layer-specific genes

The above results show that at least 450 genes, which are more than 3.4% of genes that are expressed in the cerebellum, are primarily expressed in one layer (mostly the Purkinje and granular layers). There could be many reasons for this highly structured expression pattern. For example, localized genes may reflect unique cell-type dependent biological processes, like shaping the cell morphology or controlling the connectivity between specific neuron types. Alternatively, localized expression may also reflect properties that are not necessarily cell-type specific, like processes that depend on cell size, since Purkinje cells are exceptionally large. We therefore turned to characterize the properties of localized genes, by testing their functional annotations and comparing them with the transcriptome of Purkinje-deficient mice.

#### Comparison with Purkinje deficient mice

To better characterize the properties of genes localized to the Purkinje layer, we aimed to separate genes whose expression is related to Purkinje cells from genes whose expression is related to non-Purkinje cells. We compared our study with a study by Rong and colleagues [9] who aimed to identify Purkinje-cell specific genes. Rong et al compared the cerebellar gene expression of two strains of mice: wild-type mice and 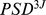 mice which have a mutation in the gene Nna1 causing them to lose their Purkinje cells by adulthood. Genes with reduced expression in the 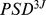 mice presumably reflect the loss of Purkinje cells.

**Figure 7:**
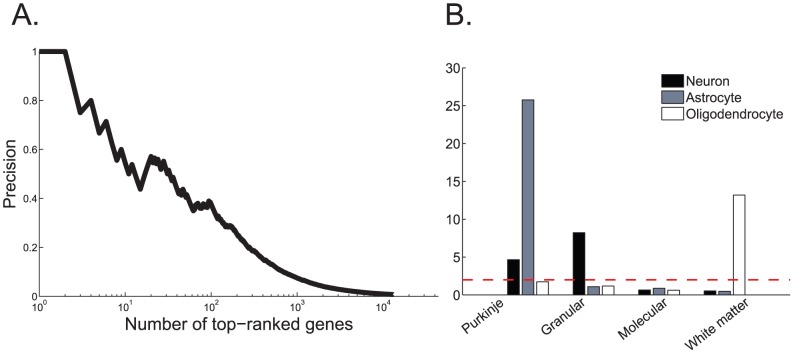
Comparison with Purkinje-deficient mice and layer enrichment for cell types (A). Comparison with Purkinje-deficient mice genes from [9]. The overlap of the set of top ranked genes that were localized to the Purkinje layer with 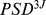 Precision is the fraction of Purkinje-localized genes that are found in 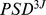 mice. (**B**) Enrichment for cell type specific markers, taken from Cahoy [7]. For each layers enrichment for cell type was tested using a hypergeometric test. The dashed red line corresponds to p-value at random. As expected, the white matter was enriched for oligodendrocyte markers and the granular layer is enriched for neuronal markers. Interestingly, Purkinje layer genes show a strong enrichment for astrocytes markers.

We compared the list of genes that we predicted to localize to the Purkinje-layer with a list of 203 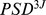 genes whose expression decayed by more than 50% as provided by [9]. We sorted the predicted genes by the classifier margin, treated the 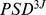 list as positives, and computed the precision at the 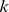 top-ranked genes. Figure 7 A shows that the top ranked predicted genes have high overlap with the 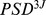 list, reaching 33% at the top 10.

The cross-comparison between the two sets reveals genes that are localized to the Purkinje-layer, but are not Purkinje-cell related Figure 7A. This may include genes that are expressed in non-Purkinje cells such as Bergmann glia. The cross-comparison also reveals genes whose expression is affected by the deficient Purkinje cells, but are not localized to the Purkinje layer. These may include genes that are expressed in the dendritic arbors of Purkinje cells, or other genes that are not layer- specific but were affected by the deficiency of the Purkinje cells. Finally, for those genes that are detected to be both Purkinje-layer related and Purkinje- cell related, the cross-comparison strengthens their link to Purkinje cells.

#### Functional annotation

As the next step, we studied known functions of the genes that were localized to the four classified layers. We used Gene Ontology (GO) annotations to find the biological processes that are over-represented in the resulting gene sets for each layer.

As expected, genes localized to the white matter layer showed enrichment for myelination. More interesting was the enrichment for neurogenesis, which is also known to take place in the white matter [25]. The Purkinje layer was enriched for lipid metabolic processes and more general processes, such as *oxidation/reduction*. Full lists of enriched categories are provided in Tables 3, 4, 5, and 6.

**Table 3:** Functional enrichment of genes localized to the white matter.

GO.ID	term	# of annotated genes	# of significant	FDR *q*-value
0042552	myelination	21	2	0.00045
0006665	sphingolipid metabolic process	36	2	0.00134
0008654	phospholipid biosynthetic process	49	2	0.00248
0022008	neurogenesis	267	3	0.00703

For each gene ontology category (GO), shown are the GO ID, the name of the category, the number of white-matter genes that are annotated with the GO category, the number of genes predicted to be in the white matter layer in this category and the FDR *q*-value computed following the method of Benjamini and Hochberg's [33]. Enriched categories indeed include white-matter functions (myelination) but also regulation of neurogenesis which was also found to take part in the cerebellum white matter [25].

**Table 4:** Functional enrichment of genes localized to the Purkinje layer.

GO.ID	term	# of annotated genes	# of significant	FDR *q*-value
0006816	calcium ion transport	86	12	9.5e-05
0006937	regulation of muscle contraction	22	5	0.0012
0030900	forebrain development	73	9	0.0018
0006629	lipid metabolic process	407	28	0.0018
0055114	oxidation reduction	375	26	0.0023
0007264	small GTPase mediated signal transduction	263	19	0.0057
0050767	regulation of neurogenesis	61	7	0.0083

Columns as in table 3. To support their large cell body and vast dendritic tree, the Purkinje cells have rapid metabolic processes, in agreement with the enrichment of *lipid metabolic process*.

**Table 5:** Functional enrichment of genes localized to the granular layer.

GO.ID	term	# of annotated genes	# of significant	FDR *q*-value
0016192	vesicle-mediated transport	279	14	0.00036
0009966	regulation of signal transduction	317	12	0.00952

Columns as in table 3.

**Table 6:** Functional enrichment of genes localized to the molecular layer.

GO.ID	term	# of annotated genes	# of significant	FDR *q*-value
0050804	regulation of synaptic transmission	49	2	0.0024
0006457	protein folding	76	2	0.0059

Columns as in table 3.

The cerebellar cortical layers are comprised of distinct types of neurons and glia. We asked whether the genes expressed in the different layers are associated with specific cell types. To answer this question, we used lists of genes that were found to be enriched in three major cell types; neurons, astrocytes and glia [7]. Enrichment was determined by isolating these cell populations using *Fluorescence-Activated Cell Sorting* (FACS) and quantifying their expression using microarrays. Genes with a 20-fold and up over-expression levels were defined as cell-type specific markers. The lists of cell type markers include 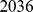 genes for neurons, 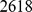 for astrocytes and 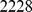 for oligodendrocytes. We tested for enrichment of these markers in our results, using the entire genome as background. Results are presented in Figure 7B. As expected, genes that were found to be expressed in the cerebellar white matter show a strong enrichment signal for oligodendrocytes. The granular layer, which contains large amounts of densely packed granule cells, indeed shows enrichment for neuron-related genes. The Purkinje cell layer, which is defined by the cell bodies of Purkinje neurons shows, interestingly, a strong enrichment signal for glia cells, notably astrocytes. This could be explained by the specialized astrocytes that occupy this layer, the Bergmann glia, and also by the astrocyte processes derived from cells located in the upper granular layer, covering the Purkinje cell bodies [26]. Oligodendrocytes are also known to be localized close to the Purkinje cells [26]. This fact can account for the enrichment of this cell type in the Purkinje cell layer.

**Figure 8:**
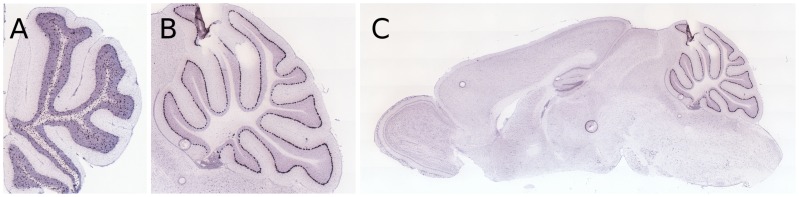
Examples of novel genetic markers. Non masked ISH images showing cerebellar expression of Map2k6 (**A**) and Fam107b (**B**). These are the raw images before the application of the expression mask. The expression of actual labeled mRNA target transcripts is marked with dark spots. (**C**) Whole-brain ISH image for Fam107b. Fam107b shows strong, highly localized expression in the Purkinje layer of the cerebellum.

Finally, we used the localization predictions to identify novel genetic markers for the different cerebellar layers. Out of the hundreds of new markers, here we describe two examples of genes that were top-ranked by our classifier in two layers. The first, Mitogen-activated protein kinase kinase 6 (Map2k6), was the first-ranked gene in the white matter. Its cerebellar expression pattern, depicted in Figure 8A, shows it is indeed clearly localized to the white matter. Map2k6 is a member of the Map kinase signal transduction pathways, and is thus involved in cell proliferation and growth. It has been shown that the human ortholog of Map2k6 is activated in the cerebellum in response to calcium, triggering a signaling pathway which results in the expression of genes responsible for the survival of newly differentiated neurons [27]. Therefore, it is not surprising to find it in the white matter of the cerebellum, and yet this expression pattern was never previously demonstrated. While Map2k6 is a relatively well-studied gene, the second example we discuss, Fam107b (3110001A13Rik), ranked 6th by the Purkinje layer detector, has little to no associated information. This gene shows a strong, localized expression in the Purkinje layer (Figure 8 B). Moreover, its expression is also largely specific to the cerebellum (Figure 8 C).

#### Detecting layers in the developing mouse brain

To demonstrate the potential of automated classifiers, we further applied the trained detectors to find genes with localized expression over development, that could reflect temporal functional specificity. We applied our Purkinje layer detectors to ISH images from the Allen developing mouse brain atlas http://developingmouse.brain-map.org/. The images are derived from brains of mice in several developmental stages. Here we applied the classifier to postnatal stages: P4 (postnatal day 4), P14, P28 and P56. Even though our classifiers were trained on adult mice they were able to find genes expressed in the Purkinje layer with high accuracy (P28 - 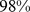 out of 69 positives, P14 - 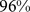 out of 56 positives). Using our classifier we found genes that are switched on and genes that are switched off in cerebellar layers during postnatal development. For example, the gene Doc2b is highly expressed in the adult Purkinje layer, but is only “switched on” at P14. Doc2 proteins act as 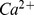 sensors and trigger spontaneous neurotransmitter release in the adult mice Purkinje cells [28]. Doc2b has also been suggested to be involved in embryonic neural development [29]. The fact that Doc2b is only switched on during late postnatal development in the cerebellum is congruent with the fact that the cerebellum becomes developed and functional only after birth, and suggests that this gene plays a role in Purkinje layer development and function. The full ranked results are also available in our site at http://chechiklab.biu.ac.il/~lior/cerebellum.html.

## Discussion

We described a machine vision approach for localizing genes to specific layers in the cerebellum. Using a small number of known cell-type markers, we trained classifiers based on visual features in ISH images of these genes and used the classifiers to detect other genes that exhibit similar localization patterns. The area under the ROC curve (AUC) of all four classifiers, trained for Purkinje, granular, molecular and white-matter layers, was higher than 0.94 on held-out data. Furthermore, when the predictions are evaluated on the full genome using human inspection, the Purkinje and granular classifiers achieved 98% accuracy over their top 250 ranked predicted genes.

Two factors contributed to the high classification accuracy of this approach. First, we extracted features from ISH images at multiple resolutions, capturing both texture and coarser structure in their features. Second, we combine multiple image predictions to a single gene-level decision. Together, these two factors reduced the classification error by over 50 percent compared to a naive classifier.

The fraction of genes whose expression is localized is surprisingly high: About 3.4% of the genes that are expressed in the cerebellum exhibit an expression pattern that was strongly localized to one layer. Functional enrichment suggest that part of this effect is due to the unusual shape and size of the Purkinje cells, leading to high expression of lipid metabolism genes. Interestingly, by comparing the genes localized to the Purkinje layer, with genes detected in Purkinje deficient mice [9], we find that many Purkinje-layer genes are not necessarily expressed in Purkinje cells. This result, together with the fact that Purkinje-layer genes are associated with astrocytes, suggests that the transcriptome of Bergmann astrocytes, which reside in the Purkinje layer, has a wider range of specifically expressed genes than previously suspected. The full list of Bergmann glia specific genes is provided in the Supporting Information. This list could be used to further understand the unique properties of Bergman Glia cells.

The large fraction of genes which exhibit localized pattern of expression hints to a high level of functional specialization across different cell types in the brain. It suggests that the average transcriptome of a neural tissue is actually a very heterogeneous mix of genes, some of which are expressed in unique cell types. This is in agreement with microarray analysis of specific cortical layers in the Rhesus monkeys [5], where the variability of the transcriptome across layers is significant.

The approach described in this paper can be used in conjunction with other approaches to improve our understanding of how the transcriptome changes between different types of neurons and glia cells. For instance, the transcriptome of transgenic mice that lack Purkinje neurons [9] can be used to further delineate the transcriptome of Bergmann and Purkinje cells, both a part of the Purkinje layer.

Our approach was applied to the cerebellum where the layers have a clear and pronounced structure. Other brain regions, including the dentate gyrus, the CA areas in the hippocampus or the anterior olfactory nucleus also contain laminar structures and could be analyzed in a similar way. Furthermore, it will be interesting to extend this approach to learn more refined discriminations. For example, in many brain areas astrocytes and neurons have different spatial distributions and sizes, suggesting that it may be feasible to train detectors that are sensitive to these differences. This could help further characterize the genetic profiles of many specific cell types across the brain.

## Methods

### Data and labeling procedure

We used gene expression images measured using in situ hybridization (ISH). The images were collected by the Allen Brain Institute and published online as the Allen Brain Atlas (ABA) [11] available at http://mouse.brain-map.org.

To measure the expression of a target gene, ISH uses fluorescently labeled DNA sequences that are complementary to the target gene RNA. These DNA probes are cloned and applied to each brain slice. The complementary probes hybridize to the target RNA sequence inside the cells, while the non bound probes are washed away. This fluorescent labeling captures the spatial pattern of expression of a target gene across the brain. The quality of this approach was quantified in [30], showing mostly agreement with microarray data. ISH measurements were gathered for the adult mouse full genome, covering 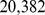 genes. For each gene, mice brains were dissected into 100 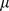
m thick slices in sagittal and coronal sections, and the slices were fluorescently labeled and imaged. The outcome of this process is a set of images for each gene showing the gene expression pattern across the whole mouse brain.

We collected a set of positive samples for four classes: *Purkinje layer*, *granular layer*, *white matter* and *molecular layer* classes. The positive samples were collected from three sources: First, we included known markers for each of the four layers. For example, we selected Calb1 as a marker of the Purkinje layer, and Plp1 for the white matter. The molecular class contained genes that are expressed in the molecular layer and also possibly in the Purkinje layers. Second, we sifted through images of 1000 random genes (ordered by gene name) and manually selected images with spatial patterns that fit the four classes. Finally, since the white matter and the molecular class had only few genes, we computed the spatial correlation of expression between the positive samples selected from the first two sources and the rest of the genome using ABA NeuroBlast [22]. We added genes whose expression in the hindbrain was highly correlated (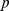-value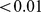) with the positive samples. Overall, the number of positive genes in each of our classes was 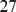, 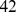, 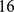 and 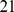 for Purkinje, granular, molecular and white matter. We also collected a set of 300 randomly selected genes (632 images) to act as a negative set for each class. For each gene we then collected all its ISH images covering the area of 600–1200 microns medial to the most lateral cut. The number of images per gene varied considerably: 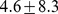 in Purkinje genes, 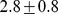 in granular, 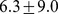 in molecular, and 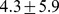 in white matter.

All together, a total of 433 genes and 1112 images were used in the labeled training set. Also, 13361 genes and 31321 unlabeled images were used in genome-wide analysis.

### Preprocessing and feature extraction

We used masked gene expression images available from ABA as RGB images. We transformed the RGB triplet at every pixel into a scalar intensity value using the *heat* color scale. Images that were completely black (no expression detected) were excluded. This happens, for example, when the sampled gene is not active in the cerebellum or when gene activity was too low to be detected by ISH. We downsampled every image at multiple resolution to capture structures at multiple scales. We used downsampling factors of 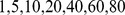.

**Figure 9:**
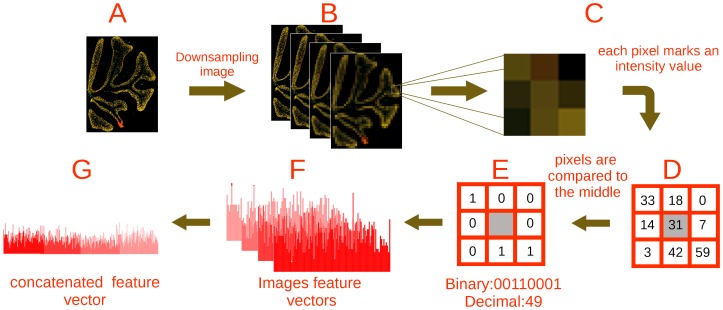
Representing expression images using LBP features. (**A–B**) The masked gene expression image is downsampled at multiple resolutions yielding several sampled images. The color marks the expression intensity of the gene at that point (**C**). The numerical level of expression at the 8 neighbors (**D**) is compared to the expression at the center, yielding an 8-bit binary word (**E**). All binary patterns from each image are collected, and their histogram is computed (**F**). All histograms are then concatenated into one - the image feature vector (**G**).

As feature vector we used local binary patterns (LBP, [12]). At every scale the LBP representation computes an 8-bits signature at every pixel of the image (Figure 9B), by comparing the pixel intensity to the intensities of its 8 circular neighbors (Figure 9D), yielding a value of 0 for a lower intensity neighbor and 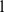 otherwise (Figure 9E). LBP signatures are then collected across the full image and their histogram is computed, yielding a 256-features vector (Figure 9F). The feature vectors of each resolution are then concatenated into a single feature vector (Figure 9G) representing the image in different scales.

In LBP, the distance from the center to the surrounding pixels could be tuned. Here we used a circle of radius 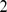 centered at each pixel (known as LBP(8,2)) which achieved superior performance in early experiments. A common variant of LBP, called uniform LBP [12], avoids collecting all 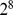 possible binary patterns separately. Instead, it merges bins that correspond to sequences (going around the center) with more than two bit flips. While in some applications uniform LBP may improve runtime and classification accuracy [12], this was not the case in our experiments, probably because ISH images have very different statistics than real world images. The total number of features per image at a specific resolution was therefore 257 (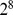 LBP features and the mean image intensity). Features were scaled to the range [0,1] by dividing by the maximal value in the histogram.

We also tested classifiers that uses SIFT features [13]. The Purkinje layer detector showed lower performance, the granular layer detector and the white matter detector performance did not change much and molecular layer detector was improved. We chose to use LBP for their ease of implementation and interpretation (Results using SIFT as features are not shown).

### Classification

**Figure 10:**
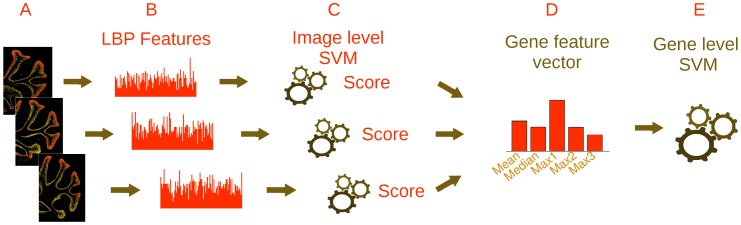
The two layered classifier. (**A**) All available images of gene Gap43 are processed (taken from different cuts or different experiments). (**B**) For each image an LBP feature vector is produced and used as input for the image level SVM. (**C**) Each image receives a confidence score from the image level SVM. (**D**) The gene's images scores are collected and a using their mean, median, first, second and third max, a gene feature vector is computed. (**E**) The gene is classified using the gene level SVM.

For image classifiers, we trained a support vector machine (SVM) to discriminate images of each layer from images of other layers. Given a new (test) image that is not used in training, each classifier can provide a “soft” decision score (Figure 10 C), based on the distance of the sample from the separating hyperplane (the margin).

To classify images, we trained an SVM using libSVM [31] with a radial basis function (RBF) kernel. We choose SVM because its inherent regularization handles well learning with a relatively small number of samples per class. We used two layers of five-fold cross-validation, one to tune the classifier parameters and the second to tune the hyper parameters. When splitting images into the train and test sets, all images of a gene were either in the train or the test set, but never in both, to avoid overfitting. We used grid search to select the best regularization hyper parameter 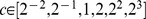, and best RBF hyper parameter 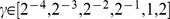. Optimal AUC was usually found to be near the middle of the regularization range and near 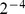 for the RBF hyper parameter.

Our dataset is highly imbalanced with many more negatives than positives for each class. To take this bias into account, we assigned different costs to false positive and false negative errors during training. This was done by setting the c+ and c− parameters in SVM, based on the relative sizes of the positive and negative sets. Also, we evaluate performance using the area under the ROC curve (AUC) a measure that is invariant to this bias.

### Pooling image information to make a single decision about a gene

Combining image scores into a single gene score is a special case of what is known as multi-instance learning (MIL). In general, MIL deals with the case were labels are assigned to a “bag” of samples, which in our case are all the images of a common gene. MIL also commonly appears in tasks like visual object recognition, where features are collected over multiple patches in a single image. The features of the instances are then pooled together using a summary statistic like the mean or maximal.

We tested three different ways to combine image scores into a decision over genes. First, we used the mean scores of all images that correspond to a certain gene. We call this approach *Mean image score*. Second, we collected the LBP features of all images that correspond to a gene, computed their average histogram, and trained a classifier using this single histogram as a feature vector. We call this approach *Mean LBP features*. Third, instead of using only a single order statistic (max, median or mean), we combined multiple order statistics of the images scores. Using the soft decision scores from all images, we trained a second classifier. This approach is referred as *Two level classifier*. We also tested a fourth approach as a baseline, treating each image independently, and making a prediction at the level of single images. This can also be viewed as classification when each gene has a single image only.

For the gene-level classifier we trained a linear SVM which receives as inputs the confidence scores (Figure 10C) of all corresponding images (their distance from the separating hyperplane). Since each gene has a different number of corresponding images, image scores were pooled using order and moment statistics (Figure 10D). Specifically, we used the mean, the median and the k-top images scores (total of 5 features). We found that best performance was achieved for 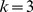. Whenever a gene had less than 3 corresponding images the lowest available value was duplicated to fill the missing values. We used again two layers of 5-fold cross-validation for tuning the SVM regularization hyper-parameter 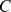.

### Comparison with NeuroBlast

NeuroBlast [22] - http://mouse.brain-map.org/ ranks in-situ images according to their spatial correlation to an image of a specific gene (the seed). We focused on correlation in the cerebellum region. For seeds we used a layer known marker (Plp1, Calb1, Neurond1 and Pvalb for the white matter, Purkinje, granular, molecular layers respectively). For each gene from our manual labeled list we collected the correlation scores of its images and computed their mean correlation score. These gene scores were used to calculate the AUC for each layer.

### Functional enrichment

GO enrichment was tested using *elim* [32], which takes into account local dependencies in the hierarchical structure of the Gene Ontology trees, and then by applying the fisher exact test to determine statistical significance of the results (compared to hypergeometric distribution). 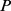-values were corrected for multiple comparisons using FDR [33].

## Supporting Information

Table S1 **Purkinje layer detector results.** The full list of genes predicted to reside in the Purkinje layer - ordered by confidence score.
(CSV)

Table S2 **Purkinje layer astrocytes.** List of genes in the Purkinje layer that are also enriched for astrocytes (Cahoy [7]).
(CSV)

Table S3 **Purkinje layer but not Purkinje cells.** List of genes in the Purkinje layer that not Purkinje cells. These genes are expressed in the Pukinje layer but do not appear in the list of Purkinje cell related genes from Rong et al [9].
(CSV)

Table S4 **Bergmann glia.** List of genes in the Purkinje layer that show enrichment for astrocytes [7] and also do not appear to be related to Purkinje cells [9].
(CSV)

Table S5 **Purkinje cells (Rong).** List of Purkinje cell related genes from [9].
(CSV)
